# Supplementation with Yellow Mealworm (*Tenebrio molitor*) Larvae Grown on Deoxynivalenol-Contaminated Substrate Improves Growth and Gut Integrity in Broilers

**DOI:** 10.3390/toxins18080341

**Published:** 2026-08-04

**Authors:** Revathi Shanmugasundaram, Klint W. McCafferty, Laharika Kappari, Xin Ye, Kelsy Robinson, Anthony E. Glenn

**Affiliations:** 1 Toxicology and Mycotoxin Research Unit, U.S. National Poultry Research Center, Agricultural Research Service, U.S. Department of Agriculture, Athens, GA 30605, USA; 2 Poultry Research Unit, Agricultural Research Service, U.S. Department of Agriculture, Starkville, MS 39762, USA; 3 Department of Poultry Science, University of Georgia, Athens, GA 30602, USA; 4 Department of Poultry Science, Mississippi State University, Starkville, MS 39762, USA

**Keywords:** larval meal, deoxynivalenol, broilers, sustainability

## Abstract

Deoxynivalenol (DON), a *Fusarium* mycotoxin frequently detected in poultry feeds, impairs broiler health by damaging gut integrity, altering immune response, and compromising growth performance. Insect-derived proteins, such as those from yellow mealworm (*Tenebrio molitor*) larvae meal (YMW), are gaining interest as an alternate protein source for broiler chickens. Hence, this study evaluated whether defatted YMW produced from larvae reared on DON-contaminated substrates can be safely included in broiler diets. A total of 400 one-day-old Cobb 700 mixed-sex broilers were assigned to four dietary treatments—(1) the control, (2) DON (15 mg/kg), (3) 2% YMW, and (4) 4% YMW—in ten replicates with 10 birds per replicate for 15 days. On day 14, performance parameters, intestinal morphology, tight-junction protein (TJP) gene expression, and CD4+ and CD8+ T-cell populations in cecal tonsils were evaluated. Data were analyzed using one-way ANOVA followed by Tukey’s multiple comparison test. DON exposure reduced body weight by 1% and body weight gain (BWG) by 1.2%, decreased the CD4+: CD8+ T-cell ratio by 80%, and reduced the villus height in the jejunum by 19.8% and ileum by 16%. DON altered the TJP expression by upregulating claudin-2 mRNA expression by 2.1-fold in the jejunum and 5.2-fold in the ileum, and it downregulated claudin-4 expression by 6.4-fold in the ileum (*p* < 0.05). Dietary inclusion of 2% or 4% YMW from DON-contaminated substrates had no negative effect on growth performance or immune parameters and maintained intestinal morphology and TJP gene expression, comparable to the control groups. In conclusion, defatted YMW produced from larvae reared on DON-contaminated substrates can be safely used as a sustainable alternative protein ingredient in broiler diets.

## 1. Introduction

The global poultry industry continues to grow rapidly in response to increasing demand for animal protein, particularly meat and eggs [1]. According to Research and Markets, the global poultry market was valued at $378.84 billion as of 2023 and is projected to grow to $487.39 billion by 2027. The total global food demand is expected to increase by 35% to 56% between 2010 and 2050, as the world population approaches 10 billion by 2050 [2]. This excessive demand poses a significant challenge to poultry producers to enhance productivity while maintaining environmental and economic sustainability [3,4]. To meet these goals, there is a need for innovative and sustainable nutritional strategies that improve feed efficiency and support poultry health.

One of the major constraints to sustainable poultry production is mycotoxin contamination of feed ingredients [5]. *Fusarium* species produce a range of mycotoxins and commonly infect cereal grains [6], such as corn and wheat, during pre- and post-harvest stages [7]. *Fusarium* species such as *F. graminearum* and *F. culmorum* are major producers of deoxynivalenol (DON) and can also synthesize zearalenone, while species like *F. verticillioides* and *F. proliferatum* are important producers of fumonisins [8]. Among these, DON is one of the most prevalent and harmful mycotoxins, compromising feed quality and adversely affecting poultry health and performance [9]. According to the US Food and Drug Administration (FDA) guidance, the advisory level for DON in poultry finished diet is 5 mg/kg of DON [10]. Beyond this limit, grain may be condemned or only usable through strategies such as kernel sorting or blending to reduce contamination [7]. Although acute toxicity typically occurs above this limit, chronic exposure to lower concentrations can still impair chicken performance and cause subclinical infections [10]. Although DON has a relatively low absorption rate in the poultry gastrointestinal tract (1–6%), it can persist through enterohepatic circulation [10]. At the cellular level, DON binds to ribosomes, inhibits protein synthesis, and activates cellular stress-signaling pathways. These effects can result in oxidative stress, altered cell signaling, reduced epithelial cell proliferation, and apoptosis [11]. Furthermore, DON disrupts intestinal epithelial cell integrity, alters tight-junction protein expression, suppresses immune response, and ultimately reduces nutrient absorption and growth performance [10]. Current mitigation strategies, such as mycotoxin binders and optical kernel sorting techniques using infrared technology, have shown promise but remain expensive and are not always fully effective [12,13]. As a result, large volumes of contaminated grain are wasted despite their potential nutritional value.

Insect-based feed ingredients have emerged as a promising solution to reduce feed waste and provide sustainable alternative protein sources [14,15,16]. Insect bioconversion offers a cost-effective alternative to mycotoxin binders and optical sorting. Because binders are expensive and optical sorting results in substantial grain loss [17], rearing insects on mycotoxin-contaminated substrates allows the conversion of otherwise low-value, mycotoxin-affected material into nutrient-rich protein with minimal input costs [18,19]. This approach not only reduces waste but also enhances agricultural sustainability by adding value to materials that would otherwise be discarded [20]. Broader applications of insect production systems further demonstrate measurable economic and environmental benefits, including a reduction in landfill waste and lower CO_2_ emissions. Consequently, insect bioconversion into poultry feeding systems offers a cheaper and more sustainable alternative to conventional detoxification methods [21,22], aligning with long-term goals of improving feed security, resource efficiency, and supporting the rapid growth of the emerging insect industry.

Yellow mealworm (*Tenebrio molitor*) larvae (YMW), which provide high-quality protein, lipids and bioactive compounds [23], have emerged as a potential alternative protein source in poultry diets [24]. These larvae are efficient bio-converters capable of transforming low-value agricultural by-products, including mycotoxin-contaminated substrates, into nutrient-rich biomass [25,26,27,28]. Importantly, several studies have shown that *T. molitor* larvae reared on DON-contaminated feed do not accumulate DON in their tissues, presenting a unique opportunity to recover nutrients from grain that would otherwise be discarded [26,29,30]. Ingested DON is either excreted in the frass or transformed through larval metabolic processes; consequently, YMW offers a promising waste-to-feed strategy that is both environmentally sustainable and nutritionally valuable [31].

Previous investigations have predominantly focused on DON accumulation and metabolic processing in mealworm larvae [29,32,33] or evaluated the effects of mealworm meal on broiler performance, including growth, nutrient retention, carcass yield, and organ indices such as relative organ weights [30,34]. However, limited research has evaluated the nutritional and immunological effects of supplementing yellow mealworm meal (YMW) produced from larvae reared on DON-contaminated substrates in broiler diets. The present study evaluates two dietary inclusion levels of defatted YMW (2% and 4%) produced from larvae reared on DON-contaminated substrates on broiler growth performance, immune parameters, or intestinal health, thereby supporting its potential use as a safe and sustainable alternative protein source in poultry diets. Therefore, the objective of this study was to determine the effects of dietary supplementation with defatted YMW produced from larvae reared on DON-contaminated feed on growth performance, gut integrity, T-cell percentage (CD4+ and CD8+ T-cell populations), intestinal morphology, and cytokine expression in broiler chickens.

## 2. Results

### 2.1. Effects of DON and Defatted YMW on the Growth Performance

On day 14, there were significant treatment effects on growth performance (*p* = 0.05) (Table 1). Birds in the DON group had a 1.0% lower body weight (BW) and a 1.2% lower body weight gain (BWG) compared with the control group. In contrast, birds supplemented with 2% or 4% YMW had BW and BWG values comparable to the control group. Mortality did not differ among treatments (*p* > 0.05).

On day 14, there were no significant treatment effects on feed intake (*p* > 0.05). The feed conversion ratio (FCR), however, differed among treatments (*p* < 0.05). The DON group exhibited a significantly higher FCR compared with the control group (*p* < 0.05). Birds supplemented with 4% YMW had a significantly lower FCR than the control group (*p* < 0.05). The 2% YMW group did not differ significantly from either the control or DON groups (Table 1).

### 2.2. Effects of DON and Defatted YMW on Cecal Tonsil and Spleen CD4+ and CD8+ T-Lymphocyte Percentages

In the cecal tonsils, significant treatment effects were observed for CD4+, CD8+, and the CD4+:CD8+ ratio (*p* < 0.05) (Table 2). Birds fed a DON-contaminated diet showed significantly lower CD4+ (3.68%) and CD8+ (9.81%) percentages compared with the control group (CD4+: 19.40%; CD8+: 20.52%), resulting in a reduced CD4+: CD8+ ratio (0.38 vs. 1.03). In contrast, supplementation with 2% YMW increased CD4+ percentages to 24.90% and similar CD8+ (21.01%) percentages to the control group, providing a greater CD4+: CD8+ ratio (1.30). Birds receiving 4% YMW also showed higher CD4+ (17.82%) and CD4+: CD8+ ratios than the DON group.

In the spleen, birds fed a DON-contaminated diet showed significantly decreased CD4+ (9.60%) and increased CD8+ percentages (36.97%) relative to the control group (CD4+: 15.58%; CD8+: 29.57%), resulting in a reduced CD4+:CD8+ ratio (0.26 vs. 0.55) (*p* < 0.05). Birds supplemented with 2% or 4% YMW showed comparable CD4+ (17.46% and 17.45%) and CD8+ (26.45% and 27.54%) percentages to the control group.

### 2.3. Effects of DON and Defatted YMW on Jejunal and Ileal Tight-Junction Protein mRNA Expression and Blood Biochemical Parameters

**Figure 1:**
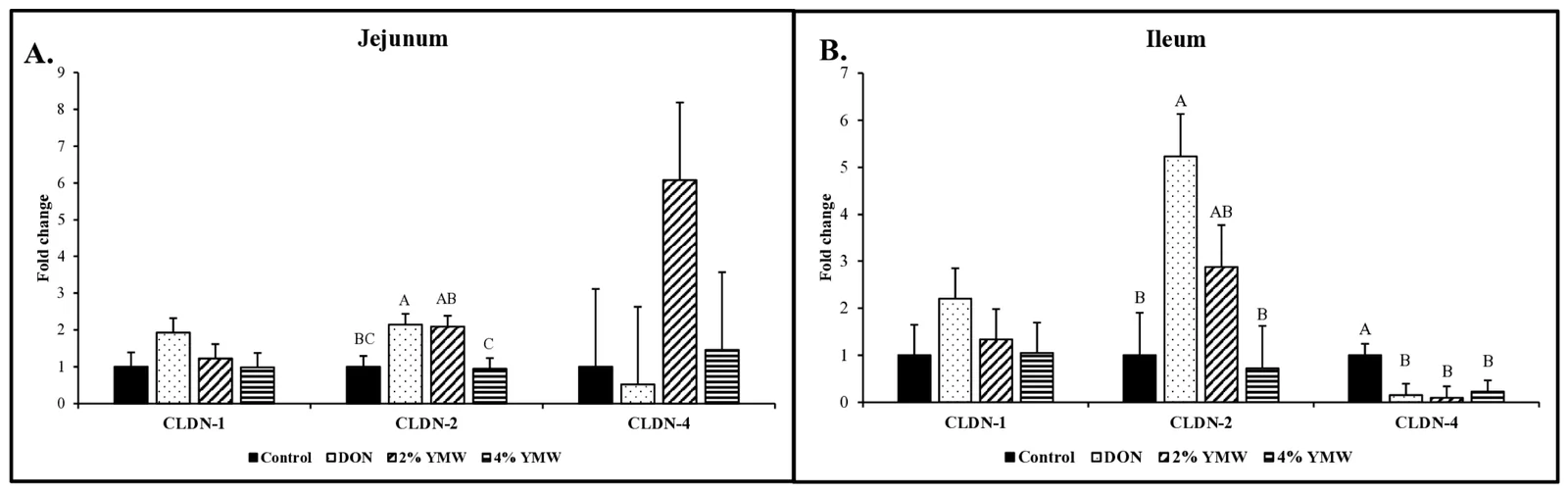
Effects of DON and defatted YMW on CLDN-1, CLDN-2, CLDN-4, and OCC mRNA expression in jejunum (**A**) and ileum (**B**) on day 14. Dietary treatments were (1) control group, (2) DON group (15 mg/kg of diet), (3) 2% YMW group (yellow meal worms reared on diets contaminated with DON at 15 mg/kg of diet), and (4) 4% YMW group (yellow meal worms reared on diets contaminated with DON at 15 mg/kg of diet). On d14, the jejunum was collected, and relative mRNA expression (qPCR) of CLDN-1, CLDN-2, and CLDN-4 was determined. Bars show least-squares means ± standard error of the mean (SEM). Bars with no common superscript differ significantly (*p* < 0.05) (*n* = 10).

Tight-junction protein mRNA expression in the jejunum and ileum under DON exposure and YMW supplementation is shown in Figure 1A,B. In the jejunum, DON significantly increased claudin-2 expression by 2.1-fold compared with the control (*p* < 0.05), whereas claudin-2 expression in birds fed 2% or 4% YMW did not differ from the control group (*p* > 0.05). Claudin-1 and claudin-4 in the jejunum were not significantly affected by dietary treatment (*p* > 0.05).

**Figure 2:**
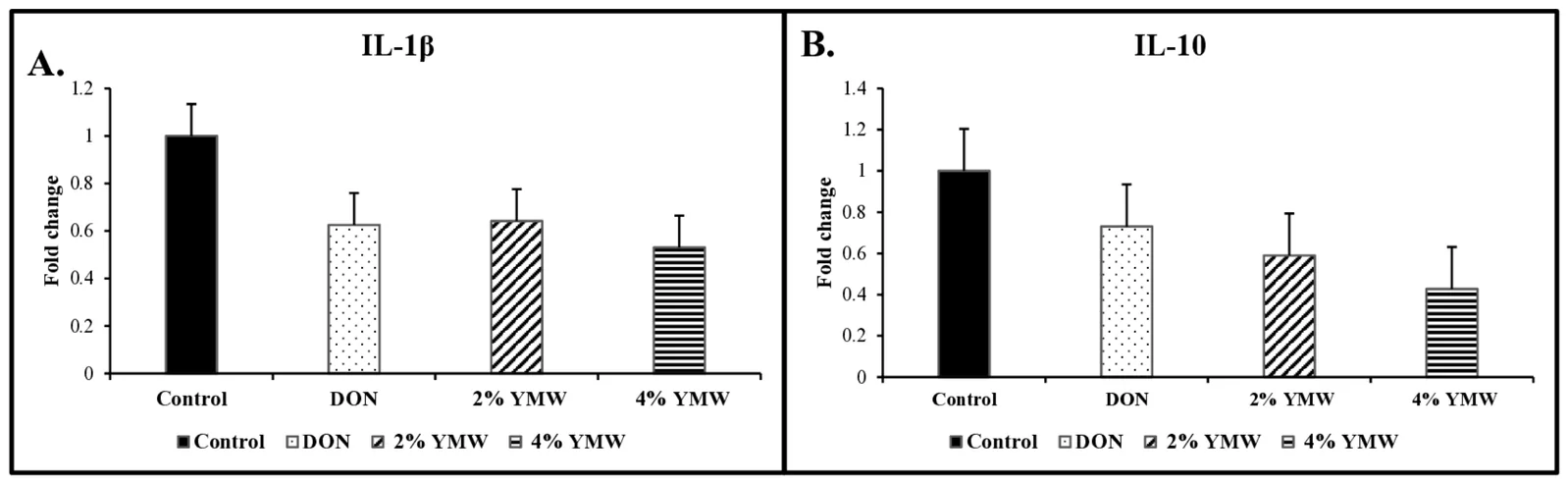
Effects of DON and defatted YMW on IL-1β (**A**) and IL-10 (**B**) mRNA expression in the ileum on day 14. Dietary treatments were (1) control group, (2) DON group (15 mg/kg of diet), (3) 2% YMW group (yellow meal worms reared on diets contaminated with DON at 15 mg/kg of diet), and (4) 4% YMW group (yellow meal worms reared on diets contaminated with DON at 15 mg/kg of diet). On d14, the ileum was collected, and relative mRNA expression (qPCR) of IL-1β and IL-10 was determined. Bars show least-squares means ± standard error of the mean (SEM) (*p* < 0.05) (*n* = 10).

In the ileum, DON significantly upregulated claudin-2 expression by 5.2-fold compared with the control (*p* < 0.05). In birds supplemented with 4% YMW, claudin-2 expression was comparable to that in the control group (*p* > 0.05). DON significantly downregulated claudin-4 expression by 6.5-fold compared with the control group. Supplementation with 2% and 4% YMW significantly decreased claudin-4 expression by 10-fold and 4.4-fold, respectively, relative to the control group (*p* < 0.05). There was no significant treatment effect on ileal inflammatory cytokine expression for IL-1β or on regulatory cytokine IL-10 expression (*p* > 0.05) (Figure 2A,B).

**Figure 3:**
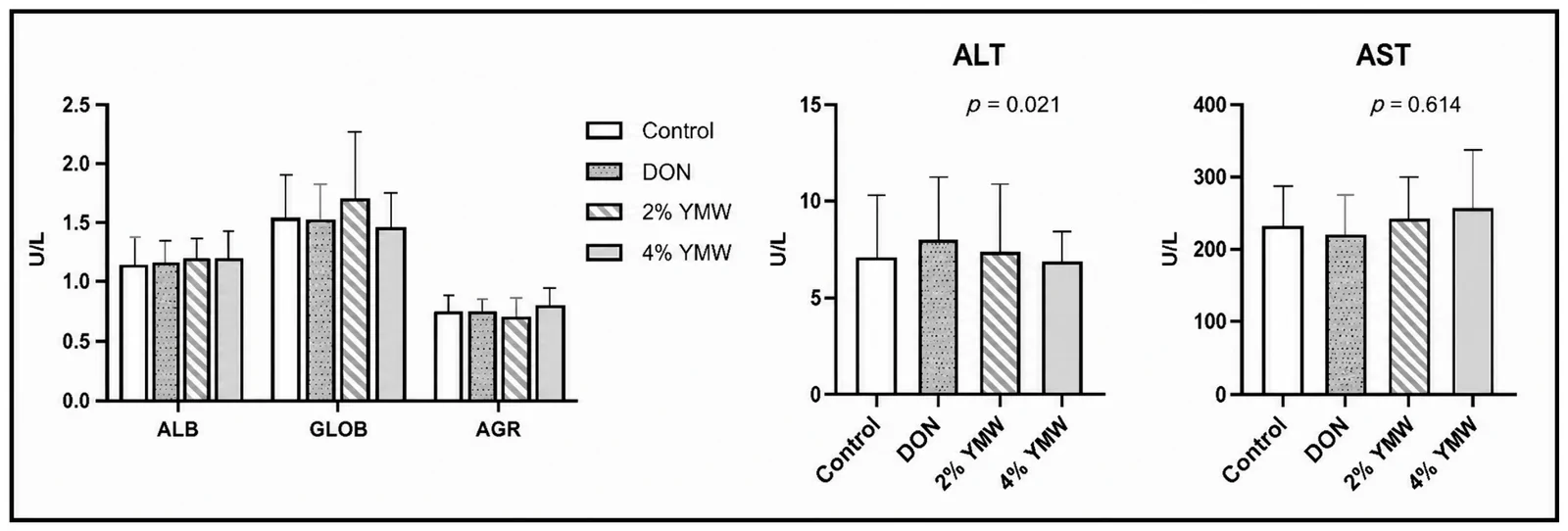
Effects of DON and defatted YMW on blood biochemical parameters on day 14. Dietary treatments were (1) control group, (2) DON group (15 mg/kg of diet), (3) 2% YMW group (yellow meal worms reared on diets contaminated with DON at 15 mg/kg of diet), and (4) 4% YMW group (yellow meal worms reared on diets contaminated with DON at 15 mg/kg of diet). On day 14, blood samples were collected and serum biochemical parameters, including albumin-to-globulin ratio (AGR), albumin (ALB), globulin (GLOB), alanine aminotransferase (ALT), and aspartate aminotransferase (AST), were analyzed. Bars show least-squares means ± standard error of the mean (SEM) (*n* = 10).

There were no significant treatment effects on blood biochemical parameters, including albumin (ALB), globulin (GLOB), the albumin-to-globulin ratio (AGR), or the liver enzymes alanine aminotransferase (ALT) and aspartate aminotransferase (AST) (*p* > 0.05) (Figure 3).

### 2.4. Effects of DON and Defatted YMW on Jejunum and Ileum Histology

**Figure 4:**
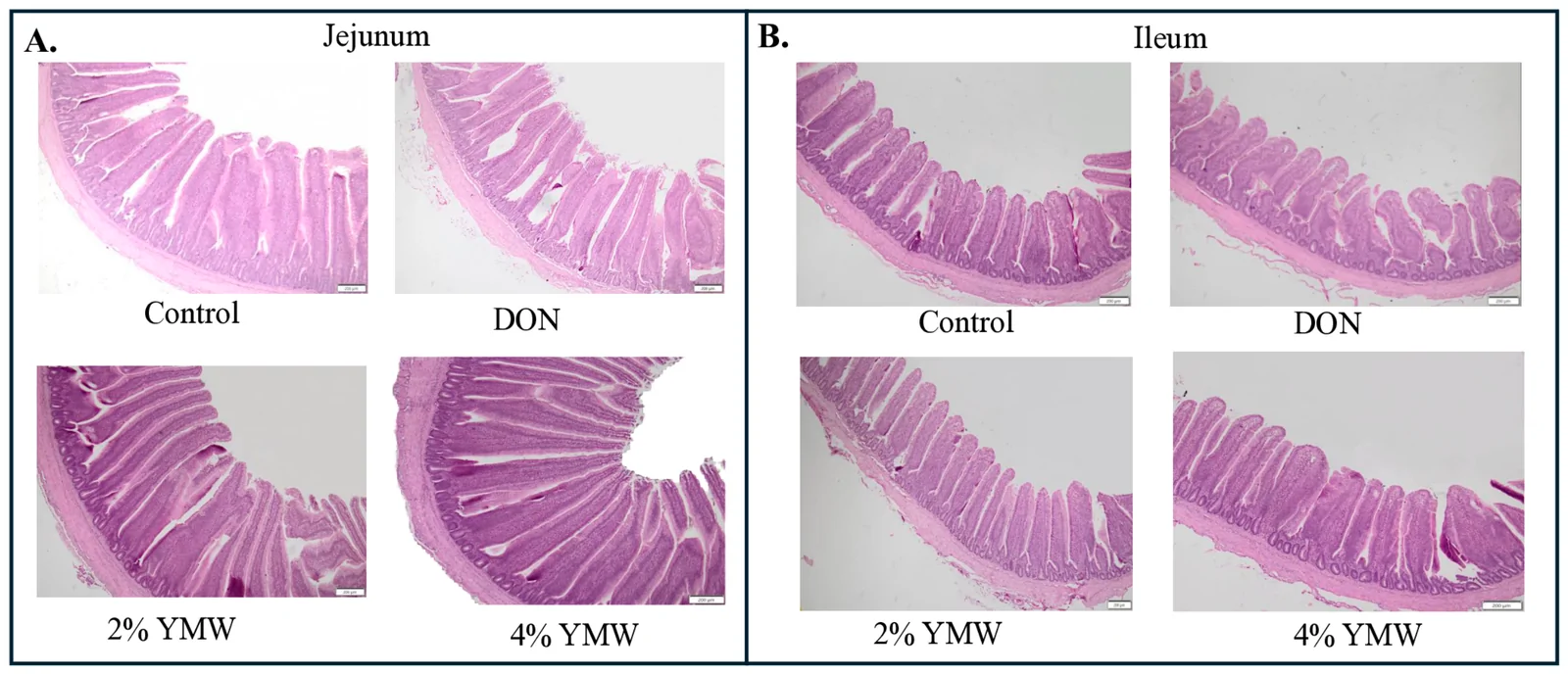
Effects of DON and defatted YMW on the jejunal and ileal histomorphology on day 14. (**A**) Hematoxylin and eosin-stained jejunal histomorphology on day 14. (**B**). Hematoxylin and eosin-stained ileal histomorphology on day 14. Scale bar = 200 μm. Dietary treatments were (1) control group, (2) DON group (15 mg/kg of diet), (3) 2% YMW group (yellow meal worms reared on diets contaminated with DON at 15 mg/kg of diet), and (4) 4% YMW group (yellow meal worms reared on diets contaminated with DON at 15 mg/kg of diet).

There was a significant treatment effect on jejunal villus height (VH) (*p* < 0.05) (Table 3) (Figure 4A). DON exposure impaired jejunal morphology, reducing VH by 20% compared with the control group (*p* < 0.05). In contrast, birds supplemented with 2% or 4% YMW showed increased VH, which were 18% and 17% higher than the control group, respectively. There were no significant treatment effects observed for jejunal crypt depth (CD) or the VH: CD ratio (*p* > 0.05).

Similar effects were observed on the ileal VH (Table 3). DON exposure reduced ileal VH by approximately 16% compared with the control group (*p* < 0.05) (Figure 4B). Birds supplemented with 2% or 4% YMW showed a higher VH than the control group, with the greatest response observed in the 2% YMW group. No significant treatment effects were observed for ileal crypt depth (CD) or the VH: CD ratio (*p* > 0.05).

## 3. Discussion

The present findings provide important evidence that YMW from larvae reared on DON-contaminated substrates is physiologically safe for broilers. Although previous work has shown that DON does not accumulate in mealworm tissues [32,35], the consequences of feeding such meal to poultry have not been evaluated completely. In the current study, broilers fed 2% or 4% YMW showed normal growth performance, T-cell profiles, intestinal morphology, and cytokine expression, indicating that the biological quality of the insect meal was not compromised by the contaminated rearing substrate. This is particularly relevant because DON contamination results in substantial grain losses in commercial production systems. The ability to convert downgraded, DON-contaminated grain into a nutritionally valuable protein ingredient without negative effects on poultry health offers a practical strategy for reducing feed waste while supporting sustainable protein sourcing.

Exposure to DON at 15 mg/kg for 14 days significantly reduced BW and BWG and impaired feed efficiency in broiler chickens. These results are consistent with previous reports of dose-dependent reduction in BW and BWG in broilers exposed to DON 2.5 mg/kg, 5 mg/kg, and 10 mg/kg for five weeks [36]. A recent study from our lab reported that chronic exposure to *Fusarium* mycotoxins decreased BWG by up to 17% and numerically increased FCR by 11 points. Such a reduction in BWG is associated with impaired nutrient digestibility and altered intestinal morphology in broiler chickens [10]. In contrast, YMW supplementation improved performance in a dose-dependent manner. Supplementation with 2% YMW showed an increase in BWG by 0.3% and decreased FCR by three points, while birds receiving 4% YMW showed an increase in BWG by 0.9% and a decreased FCR by six points, compared with the control group (FCR: 1.264). These findings are consistent with [37], who reported that dietary inclusion of YMW improved growth performance, with 5% YMW inclusion showing about a 12% increase in final body weight, whereas higher inclusion levels (10–15%) lowered body weight gain and reduced feed efficiency, likely due to the greater amounts of chitin, exo-skeletal material, and other indigestible components typical of mealworm meal, which can limit nutrient utilization. In the present study, the results suggest that up to 4% YMW inclusion optimizes growth performance, potentially through improved gut health and nutrient utilization. Chitin and its derivatives can function as prebiotic, immune-modulatory, and gut-barrier-supportive molecules, promoting beneficial microbial populations and enhancing mucosal integrity [38]. Additionally, antimicrobial peptides and cuticle-associated compounds in YMW may contribute to improved nutrient utilization [39]. Although high dietary chitin can prevent digestibility, the levels present at up to 4% inclusion appear to fall within a beneficial threshold that stimulates gut function without creating an excessive indigestible load. YMW derived from DON-contaminated substrates has been shown to retain minimal DON carryover, as mealworms efficiently excrete the toxin via frass [40,41], supporting the safety and efficacy of YMW produced under such conditions.

The intestinal morphology of the small intestine, particularly villus height, crypt depth and VH:CD ratio, reflects absorptive capacity [42] and overall gut health [43]. In the present study, DON exposure significantly reduced VH in both the jejunum (707.7 µm vs. 882.9 µm in control) and ileum (520.53 µm vs. 619.41 µm in control), corresponding to approximately 19.8% and 16.0%, respectively. Similar findings were reported in broilers exposed to DON at 10 mg/kg feed for a 35-day period, where decreased duodenal and jejunal villus heights and a reduced VH:CD ratio were found across all intestinal segments [44]. In contrast, supplementation with 2% and 4% YMW increased jejunal villus height by 46–48%, while it increased ileal villus height by 25–29%, compared with those of the controls. Previous studies have shown that supplementation of 2.5–5.0% *Tenebrio molitor* meal improves growth performance without adverse effects on intestinal morphometrics [45] and carcass characteristics of broiler chickens [30]. Additionally, YMW inclusion in the chicken diet increased the crude protein and dry matter retention, and this is most likely due to the chitin present in insect meals, which can function as a prebiotic to selectively promote beneficial bacterial taxa associated with SCFA production, contributing to improved villus architecture and epithelial barrier function [46]. Beyond chitin, mealworms contain endogenous antimicrobial peptides that can help limit pathogenic bacterial proliferation and reduce intestinal inflammation, thereby promoting a more favorable environment for nutrient absorption [22,47]. The lipid fraction of YMW, which includes medium-chain fatty acids such as lauric acid, may further contribute to gut health by limiting pathogenic bacteria and providing an efficient energy source for intestinal epithelial cells [39]. YMW has also been shown to positively modulate digestive enzyme activity, which supports improved protein and dry matter digestibility [48,49,50]. Together, these bioactive properties likely explain the enhanced nutrient utilization observed at the 4% inclusion level.

The adaptive immune system activation depends on the activation and differentiation of CD4+ and CD8+T cells [51], and their ratio serves as a reliable indicator of immune status and potential dysfunction [52,53]. At the cellular level, DON exerts immunotoxic effects by inducing immune cells’ apoptosis through altered intracellular signaling pathways [54], ultimately reducing immune cell populations [55]. In this current study, DON decreased the CD4+:CD8+ ratio by 56% in cecal tonsils, indicating immunosuppression. These findings are consistent with a previous study, where broilers exposed to *Fusarium* toxins [fumonisins (33 mg/kg) and DON (3 mg/kg)] had significant reductions in CD4+ and CD8+ T cells in the cecal tonsils and CD4+ T cells in the spleen [10]. In the present study, supplementation with 2% and 4% YMW resulted in a CD4+:CD8+ ratio comparable to that of the control group. This effect is most likely attributable to the immune-modulating compounds in YMW, including chitin, medium-chain fatty acids such as lauric acid, and insect-derived antimicrobial peptides, which collectively enhance mucosal immunity and maintain immune balance under normal physiological conditions rather than directly modulating T-cell responses [56,57,58].

Cytokine analysis in the ileum revealed no significant treatment effects on IL-1β or IL-10 expression (*p* > 0.05). The ileum was selected as the target tissue because it represents the primary site of nutrient absorption and, therefore, the intestinal region with the greatest direct exposure to dietary components, including mycotoxin-contaminated substrates. As a major segment of the mucosal immune system containing Peyer’s patches and diverse immune cell populations, the ileum provides a relevant and sensitive site for detecting early immune modulation in response to dietary toxins or bioactive feed ingredients. Although DON typically induces pro-inflammatory responses, we observed a numerical reduction in ileal IL-1β expression (1.6-fold) in birds directly challenged with DON, suggesting a suppression of local mucosal immunity rather than a classical inflammatory reaction. Previous reports have similarly documented decreased IL-1β under conditions where DON impairs innate immune signaling, supporting the possibility of DON-induced immunosuppression [59,60,61]. Similarly, DON exposure numerically reduced IL-10 expression to 1.4-fold compared with the control, indicating a suppression of anti-inflammatory signaling. In contrast, dietary inclusion of 2% or 4% YMW did not alter IL-1β or IL-10 expression, even though the meal originated from DON-contaminated substrate. Because the birds were not directly exposed to DON during the feeding trial, the absence of cytokine modulation indicates that any residual DON or DON-derived metabolites in YMW were present at levels too low to elicit an intestinal immune response [62,63]. Although insect meals contain chitin, medium-chain fatty acids, and antimicrobial peptides, these compounds did not affect cytokine expression at the inclusion levels tested. These findings demonstrate that YMW produced from DON-contaminated substrate did not compromise mucosal immune function and can be considered safe at the tested inclusion levels [64,65]. Together, these findings indicate that although YMW improved immune cell ratios and supported gut integrity, these benefits were not reflected in cytokine expression, which remained unchanged relative to the control group. Further research may be needed to determine whether higher inclusion levels or longer feeding periods influence cytokine responses, but under the conditions of this study, YMW did not exert detectable effects on inflammatory or regulatory cytokine expression.

Tight junctions are critical for intestinal barrier integrity as they regulate intestinal epithelial barrier function [66,67]. In the present study, DON exposure upregulated jejunal claudin-2 (2.1-fold) and ileal claudin-2 (5.2-fold) while downregulating jejunal claudin-4 (6.4-fold) and ileal claudin-4 (1.9-fold), consistent with previous studies [10]. Claudin-2 promotes paracellular permeability and is associated with leaky gut and intestinal inflammation [68], whereas claudin-4 enhances barrier sealing [69]. Supplementation with 2% YMW showed partial restoration (2.9-fold), whereas 4% YMW normalized claudin-2 expression (0.9-fold) in the ileum, indicating improved barrier function. Similar protective effects of YMW on gut integrity have been reported in piglets [70]. These findings suggest that YMW supplementation protects gut barrier function through its bioactive compounds, which have antimicrobial and immunomodulatory properties.

The analysis of serum biochemical parameters, including ALB, GLOB, the albumin-to-globulin ratio (AGR), and liver enzymes alanine aminotransferase (ALT) and aspartate aminotransferase (AST), revealed no significant differences among the treatment groups (*p* > 0.05). These findings suggest that neither DON exposure at 15 mg/kg nor YMW supplementation at 2% or 4% inclusion levels adversely affected hepatic function or protein metabolism in broiler chickens during the 15 d trial. The absence of changes in ALT and AST indicates that liver integrity was maintained, while stable ALB and GLOB levels reflect normal protein synthesis and immune status [71,72]. This aligns with previous reports that short-term DON exposure primarily impacts intestinal morphology and immune modulation, rather than systemic biochemical markers [44,73]. Furthermore, YMW supplementation did not induce any hepatotoxic effects, supporting its safety as a functional feed ingredient. Overall, our research findings indicate that YWM supplementation improved intestinal morphology, normalized tight-junction protein expression, improved growth performance and modulated immune responses to counteract the effects of DON in broilers, either directly or indirectly. Enhanced nutrient utilization, epithelial turnover and immune regulation may all contribute to the protective effect observed in broilers, although the mechanisms still remain unclear. Future studies should focus on detecting DON metabolites in birds fed insect meal, characterizing the role of microbial fermentation of chitin derived from insects, and identifying specific bioactive lipids or peptides in YMW that influence immune health and gut barrier integrity.

In conclusion, dietary inclusion of defatted yellow mealworm meal (YMW) had no adverse effects on broiler chickens’ performance and gut health. Birds fed 2% or 4% YMW showed improved body weight gain, a reduced feed conversion ratio, normalized jejunal and ileal villus heights, and improved tight-junction expression. These outcomes demonstrate that YMW from DON-contaminated substrate is a safe feed additive and capable of improving gut morphology, epithelial barrier regulation, and immune balance independently of DON exposure. From a sustainability perspective, the use of insect-derived proteins aligns with circular feed production systems and offers a promising alternative ingredient for poultry diets. Future research should investigate DON-metabolizing processes in mealworms reared on contaminated substrates, characterize microbial fermentation of chitin and its immunological implications, and evaluate long-term YMW supplementation across a full broiler production cycle to determine the persistence of these beneficial responses.

## 4. Materials and Methods

### 4.1. Ethics Statement

All procedures were approved by the USDA ARS Poultry Research Unit Animal Care and Use Committee (Protocol # A2021-05-013) and conducted in accordance with the Guide for the Care and Use of Agricultural Animals in Research and Teaching [74].

### 4.2. Diet Formulation and Treatments

A non-medicated corn–soybean meal-based mash diet was used as a basal diet (Table 4). The insect meal consisted of dried defatted YMW derived from yellow mealworms reared on diets contaminated with deoxynivalenol (DON) at 15 mg/kg (Table 5). The experiment was conducted for 15 days. Birds were fed with four dietary treatments: (1) the control group (no alternative protein source), (2) the DON group (basal diet supplemented with DON culture material to achieve a final concentration of 15 mg/kg, with no alternative protein source), (3) the 2% YMW group (yellow meal worms reared on diets contaminated with DON at 15 mg/kg of diet), and (4) the 4% YMW group (yellow meal worms reared on diets contaminated with DON at 15 mg/kg of diet).

### 4.3. Birds and Housing

A total of 400 one-day-old Cobb 700 mixed-sex broiler chicks were obtained from a commercial hatchery. Upon arrival, the chicks were weighed individually and randomly distributed into four treatments. Each treatment was replicated in 10 battery cages with 10 mixed-sex birds per cage, which reflects standard commercial practice and typical experimental conditions for broiler starter-phase studies. The replicate cage was considered the experimental unit for all performance measurements (*n* = 10). This study was conducted during the first 14 days post-hatch, a period when sex-related differences in growth and intestinal development are generally minimal [75,76,77]. This study was terminated on day 15. All the birds were raised under the supervision of a licensed poultry veterinarian. The cages were equipped with nipple-type waterers and thermostatically controlled heaters. The chicks had ad libitum access to feed and water throughout the experimental period. The mortality of the birds was recorded daily. All birds were euthanized by cervical dislocation, according to the American Veterinary Medical Association.

### 4.4. Growth Performance Measurement

Body weight (BW) and feed intake (FI) were recorded by cage on days 0 and 14. Mortality was recorded daily and accounted for weight gain and the feed conversion ratio (FCR). On d14, two birds from each pen (*n* = 10) were randomly selected and euthanized by cervical dislocation, and tissue samples were collected for analysis.

### 4.5. Cecal Tonsil and Spleen CD4+ and CD8+ T Lymphocyte Percentages

On day 14, the percentages of CD4+ and CD8+ T cells in the cecal tonsil and spleen cells were determined using flow cytometry [78]. In brief, single-cell suspensions from the spleen and cecal tonsils (*n* = 10) were enriched for mononuclear cells by density gradient centrifugation using Histopaque (1.077 g/mL, Sigma-Aldrich, St. Louis, MO, USA). The samples were centrifuged at 400 g for 15 min, and the cells from the resulting mononuclear cell layer were collected and washed three times in RPMI cell culture medium. Approximately 1 × 10^6^ cells were incubated for 15 min with the following antibodies: a 1:250 dilution of fluorescent-isothiocyanate-conjugated mouse anti-chicken CD4+ (Southern Biotech), a 1:450 dilution of phycoerythrin-conjugated mouse anti-chicken CD8+ (Southern Biotech, Birmingham, AL, USA), and unlabeled mouse IgG (1:200) as an isotype control. After incubation, the unbound antibodies were removed by centrifugation. The percentages of CD4+ and CD8+ cells were analyzed using a flow cytometer (Luminex, Texas, USA), and the CD8+:CD4+ ratio was calculated.

### 4.6. Jejunal and Ileal Tight-Junction Protein mRNA Expression

On day 14, 1 bird per replicate (*n* = 10) was euthanized by cervical dislocation. A portion of the distal jejunum and proximal ileum (1 cm proximal and 1 cm distal to the Meckel’s diverticulum) was collected in cryovials containing RNAlater^®^ (Ambion Inc., Austin, TX, USA) and stored at −80 °C until further analysis. The tissues were homogenized using RNAzol^®^ RT (Sigma-Aldrich, St. Louis, MO, USA) and a FastPrep^®^-96 system (MP Biomedicals, Santa Ana, CA, USA; 1200 rpm, 30 s). The homogenates were then subjected to total RNA isolation using the Directo-zol™-96 MagBead RNA kit (Zymo Research, Irvine, CA, USA) on a BioSprint^®^ 96 workstation (QIAGEN, Hilden, Germany) according to the manufacturer’s protocol. RNA concentration and purity were determined using a NanoDrop 2000 spectrophotometer (Thermo Scientific, Wilmington, DE, USA), and RNA integrity was assessed using agarose gel electrophoresis. One microgram of RNA was reverse-transcribed into cDNA using the SuperScript™ VILO™ cDNA Synthesis Kit (Thermo Fisher Scientific, Carlsbad, CA, USA). The expressions of claudin-1, claudin-2, claudin-4, interleukin-1 beta, and interleukin-10 were analyzed by subjecting cDNA to real-time PCR on a QuantStudio™ 6 Pro system using TaqMan™ Fast Advanced Master Mix (Applied Biosystems, Vilnius, Lithuania) and predesigned TaqMan™ assays (assay IDs in Table 6). Each well contained 10 µL reactions with 1 µL of diluted cDNA per well. cDNA was diluted three-fold for target genes and thirty-fold for the housekeeping gene (GAPDH). The fast-cycling conditions for all genes included an initial denaturation at 95 °C for 2 min (1 cycle), followed by 95 °C for 1 s and 60 °C for 20 s (40 cycles). Expression levels were normalized to GAPDH, and the 2^−∆∆Ct^ method was used to calculate mRNA fold change, as previously described [79], where Ct is the threshold cycle. The fold change was calculated as 2 ^(Ct Sample−housekeeping)^/2 ^(Ct Reference−housekeeping)^. The reference group was the control group.

### 4.7. Jejunum and Ileum Histology

On day 14, approximately 2 cm sections of the jejunum and ileum were collected from regions proximal and distal to the Meckel’s diverticulum. Samples were collected from 1 bird/cage (*n* = 10) from each replication and fixed in buffered formalin. Tissues were processed using a tissue processor (Sakura Finetek USA, Inc., Torrance, CA, USA) and embedded in paraffin. Paraffin blocks were sectioned at 5 μm thickness and mounted on Superfrost slides (Thermo Fisher Scientific, Waltham, MA, USA). Cross-sections were stained with hematoxylin and eosin and examined using the cellSens Imaging software, version 4.3.1 (Olympus America, Central Valley, PA, USA) to evaluate intestinal morphology, including villus height, crypt depth, and the villus–crypt ratio.

### 4.8. Blood Chemical Analysis

Blood samples were collected from the brachial wing vein (*n* = 10) in heparinized syringes and stored on ice for transfer to the laboratory. The samples were centrifuged at 4000 *g* for 20 min, and the resulting plasma was decanted into 2.5 mL graduated tubes and stored at −20 °C for later chemical analyses. The serum samples were removed from the freezer, thawed and analyzed for the serum concentrations of albumin (ALB), globulin (GLOB), alanine aminotransferase (ALT), and aspartate aminotransferase (AST) using an ACE-AXCEL (Alfa Wassermann Diagnostic Tech, West Caldwell, NJ, USA) through biochemical and enzymatic rate reactions, according to the manufacturer’s instructions. The albumin-to-globulin ratio (AGR) was calculated for each sample using the data from above.

### 4.9. Statistical Analysis

The data were analyzed using a completely randomized design, with the replicate cage (*n* = 10 per treatment) serving as the experimental unit for growth performance variables. For intestinal morphology and gene expression, one bird per replicate was randomly selected for tissue collection, while the replicate cage remained the experimental unit for statistical analysis. Because chicks were obtained as a natural mixed-sex population and randomly allocated to replicates and treatments, sex was expected to be approximately balanced across treatment groups; therefore, sex was not included as a fixed effect in the statistical model. All data were evaluated for normality and homogeneity of variances prior to analysis. A one-way ANOVA (JMP Pro 18 software, Cary, NC, USA) was used to examine the effects of the insect meal on dependent variables with the cage considered as the experimental unit. When significant main effects were detected (*p* < 0.05), mean differences among treatments were compared using Tukey’s honest significant difference test. The results are presented as least-squares means ± standard error of the mean (SEM).

## Figures and Tables

**Table 1:** Effects of DON and defatted YMW on the growth performance of broilers from 1 to 14 d of age.

Parameter/Treatment	Control	DON	2% YMW	4% YMW	SEM	Treatment Effects (*p*-Value)
Body Weight (kg)	0.396 ^ab^	0.392 ^b^	0.396 ^ab^	0.399 ^a^	0.006	0.05
BW Gain (kg)	0.346 ^ab^	0.342 ^b^	0.347 ^ab^	0.349 ^a^	0.006	0.05
Feed Intake (kg)	0.44	0.45	0.43	0.42	0.011	0.29
FCR (kg:kg)	1.26 ^ab^	1.30 ^a^	1.23 ^ab^	1.20 ^b^	0.024	0.03
Mortality (%)	4.00	2.00	4.00	4.00	1.9	0.66

Dietary treatments were (1) control group, (2) DON group (15 mg/kg of diet), (3) 2% YMW group (yellow meal worms reared on diets contaminated with DON at 15 mg/kg of diet), and (4) 4% YMW group (yellow meal worms reared on diets contaminated with DON at 15 mg/kg of diet). Means within a row for a given measurement not sharing a common superscript differ significantly (*p* ≤ 0.05) (*n* = 10). Least-squares means were separated using Tukey’s honest significant difference test. Values are presented as least-squares means ± standard error of the mean (SEM).

**Table 2:** Effects of DON and defatted YMW on CD4+ and CD8+ percentages and CD4+: CD8+ ratio in cecal tonsils and spleen on day 14.

Tissue	Treatment	CD4+ (%)	CD8+ (%)	CD4+:CD8+
Cecal Tonsils	Control	19.40 ^b^	20.52 ^ab^	1.03 ^a^
	DON	3.68 ^c^	9.81 ^c^	0.38 ^b^
	2% YMW	24.90 ^a^	21.01 ^a^	1.29 ^a^
	4% YMW	17.82 ^b^	15.15 ^bc^	1.20 ^a^
	SEM	1.35	1.47	0.14
	*p*-value	<0.0001	<0.0001	0.0002
Spleen	Control	15.58 ^a^	29.57 ^b^	0.55 ^a^
	DON	9.60 ^b^	36.97 ^a^	0.26 ^b^
	2% YMW	17.46 ^a^	26.45 ^b^	0.69 ^a^
	4% YMW	17.45 ^a^	27.54 ^b^	0.65 ^a^
	SEM	0.88	1.53	0.05
	*p*-value	<0.0001	<0.0001	<0.0001

Dietary treatments were (1) control group, (2) DON group (15 mg/kg of diet), (3) 2% YMW group (yellow meal worms reared on diets contaminated with DON at 15 mg/kg of diet), and (4) 4% YMW group (yellow meal worms reared on diets contaminated with DON at 15 mg/kg of diet). Means within a row for a given measurement not sharing a common superscript differ significantly (*p* ≤ 0.05) (*n* = 10). Least-squares means were separated using Tukey’s honest significant difference test. Values are presented as least-squares means ± standard error of the mean (SEM).

**Table 3:** Effects of DON and defatted YMW on the jejunal and ileal histomorphology on day 14.

Intestinal Segment	Treatment	Villus Height (µm)	Crypt Depth (µm)	VH:CD Ratio
Jejunum	Control	882.9 ^b^	131.84	8.11
	DON	707.7 ^c^	131.93	5.78
	2% YMW	1046.0 ^a^	130.08	8.52
	4% YMW	1035.7 ^a^	125.03	8.67
	SEM	31.61	12.21	0.93
	*p*-value	<0.0001	0.9754	0.1161
Ileum	Control	619.41 ^a^	95.62	6.66
	DON	520.53 ^c^	113.48	4.88
	2% YMW	673.42 ^a^	118.14	6.39
	4% YMW	651.82 ^a^	105.75	6.66
	SEM	22.41	9.86	0.59
	*p*-value	0.0002	0.4208	0.1177

Dietary treatments were (1) control group, (2) DON group (15 mg/kg of diet), (3) 2% YMW group (yellow meal worms reared on diets contaminated with DON at 15 mg/kg of diet), and (4) 4% YMW group (yellow meal worms reared on diets contaminated with DON at 15 mg/kg of diet). Jejunal and ileal sections were evaluated for villus height (VH), crypt depth (CD), and villus height-to-crypt depth ratio (VH: CD). Means within a row for a given measurement not sharing a common superscript differ significantly (*p* ≤ 0.05) (*n* = 10). Means were separated using Tukey’s honest significant difference test. Values are presented as least-squares means ± standard error of the mean (SEM).

**Table 4:** Ingredient and nutrient composition of the starter basal diet.

Ingredient, % “As-Fed”	Starter Basal
Corn	58.17
Soybean meal	37.88
Soybean oil	3.23
L-lysine	0.22
DL-methionine	0.33
Threonine	0.17
Calculated nutrient content (%, unless otherwise indicated)	
AME, kcal/kg	3147
Crude protein	22.6
Digestible Lys	1.26
Digestible Met	0.93
Digestible Thr	0.91
Ca	0.13
Non-phytate P	0.08
Na	0.02

AME = apparent metabolizable energy; Ca = calcium; Na = sodium; digestible Lys = digestible lysine; digestible Met = digestible methionine; digestible Thr = digestible threonine; non-phytate P = non-phytate phosphorus. All values are expressed on an as-fed basis, and nutrient contents were calculated from standard ingredient composition values.

**Table 5:** Ingredient composition of all dietary treatments ^1^ and nutrient composition of the control diet fed to Cobb 700 mixed-sexed broilers from 1 to 15 d of age.

	Dietary Treatments			
Ingredient, % “as-fed”	(1) Control	(2) DON	(4) 2% YMW	(5) 4% YMW
Starter basal	95.49	93.80	93.49	91.49
Yellow mealworm meal	---	---	2.00	4.00
Dicalcium phosphate	1.70	1.70	1.70	1.70
Calcium carbonate	1.22	1.22	1.22	1.22
Titanium dioxide	0.50	0.50	0.50	0.50
Sodium chloride	0.36	0.36	0.36	0.36
Choline chloride, 70%	0.33	0.33	0.33	0.33
Vitamin and mineral premix ^1^	0.25	0.25	0.25	0.25
Sodium bicarbonate	0.16	0.16	0.16	0.16
Deoxynivalenol culture ^2^	---	1.69	---	---
Calculated nutrient content (%, unless otherwise indicated) ^3^				
AME, kcal/kg	3005	---	---	---
Crude protein	21.55	---	---	---
Digestible Lys	1.20	---	---	---
Digestible Met	0.88	---	---	---
Digestible Thr	0.87	---	---	---
Ca	0.86	---	---	---
Non-phytate P	0.43	---	---	---
Na	0.21	---	---	---

YMW = insect meal consisted of dried defatted yellow mealworm meal derived from yellow mealworms reared on diets contaminated with deoxynivalenol at 15 mg/kg. ^1^ Vitamin and mineral premix supplied per kilogram of diet: retinol, 7716 IU; cholecalciferol, 2756 IU, tocopherol acetate, 17 IU; cyanocobalamin, 0.011 mg; menadione, 0.83 mg; riboflavin, 6.61 mg; pantothenic acid, 6.61 mg; thiamine, 1.10 mg; niacin, 27.6 mg; pyridoxine, 1.38 mg; folic acid, 0.69; biotin, 0.03 mg; choline, 386 mg; Mn (manganese sulfate), 100 mg; Zn (zinc sulfate), 100 mg; Fe (ferrous sulfate), 50 mg; Cu (copper chloride and sulfate), 11.3 mg; Se (sodium selenite), 0.15 mg; and I (calcium iodate), 1.5 mg. ^2^ Deoxynivalenol culture material was included in the DON group at the expense of the starter basal ingredient to achieve a calculated concentration, 15 mg/kg, of DON in the finished feed. ^3^ Calculated nutrient content of the control diet is only displayed because the nutrient values of the alternative protein ingredients and DON culture material used in dietary treatments 3 and 4 and treatment 2, respectively, were not known, so test ingredients were included at the expense of the starter basal.

**Table 6:** The assay IDs and catalog numbers of primers used for relative gene expression analysis.

Gene	Assay ID *	Catalog Number	Reporter Dye	Accession Number
Glyceraldehyde 3-phosphate dehydrogenase (GAPDH)	Gg03346982_m1	4331182	FAM	NM_204305.1
Claudin-1 (CLDN-1)	Gg03343927_m1	4351372	FAM	NM_001013611.2
Claudin-2 (CLDN-2)	Gg03313695_m1	4351372	FAM	NM_001277622.1
Claudin-4 (CLDN-4)	Gg07168536_s1	4351372	VIC	AY435420.1
Interleukin-1β (IL-1β)	Gg03347157_g1	4351372	FAM	Y15006.1
Interleukin-10 (IL-10)	Gg03358689_m1	4351372	FAM	NM_001004414.4

* All assays were predesigned TaqMan^®^ Gene Expression Assays (Applied Biosystems, Thermo Fisher Scientific). For qPCR, cDNA was diluted threefold for target genes and thirtyfold for GAPDH; 1 µL of diluted cDNA was added per 10 µL reaction.

## Data Availability

All data supporting the findings of this study are included within this manuscript. Further inquiries can be directed to the corresponding author.
